# Cohort Differences in Self-Objectification

**DOI:** 10.1093/geroni/igab046.215

**Published:** 2021-12-17

**Authors:** Sydney Tran

**Affiliations:** Oregon State University, Corvallis, Oregon, United States

## Abstract

Sexual objectification socializes women to engage in self-objectification—the tendency to view one’s body as an object to be used by others and evaluating one’s value in terms of attractiveness to others (Noll & Fredrickson, 1998)—and leads to negative psychological consequences. As women age, their bodies move further away from the thin ideal (Guo, Zeller, Chumlea, & Siervogel; 1999) potentially making them more susceptible to body i concerns and dissatisfaction. However, may also begin using selection, optimization, and compensation (SOC) strategies, countering the impacts of sexual objectification, and promoting successful aging. We compared self-objectification between women in early adulthood (N = 132, M = 20.93) and women in late middle age or late adulthood (N = 86, M = 67.83). Results showed that older women had significantly lower levels of self-objectification than younger women. Our findings support the idea the SOC strategies protect against the consequences of sexual objectification.

